# Supplementary material for: Cystic Fibrosis Clinical Isolates of Aspergillus fumigatus Induce Similar Muco-inflammatory Responses in Primary Airway Epithelial Cells
Source: Pathogens. 2021 Aug 13;10(8):1020. doi: 10.3390/pathogens10081020 (PMC8399118; doi:10.3390/pathogens10081020)
Supplement: Supplementary file 1 [file pathogens-10-01020-s001.zip › pathogens-1302880-supplementary.pdf]

Table S1: Identification using MALDI-TOF MS biotyper based on Bruker's broth-extraction method.

Isolate ID	Grade	Organism (best match)	Log score	Organism (second best match)	Log score
ATCC (#46645)	(+++) (A)	*Aspergillus fumigatus*	2.443	*Aspergillus fumigatus*	2.438
CFA001	(+++) (A)	*Aspergillus fumigatus*	2.366	*Aspergillus fumigatus*	2.143
CFA002	(+++) (A)	*Aspergillus fumigatus*	2.347	*Aspergillus fumigatus*	2.280
CFA003	(+++) (A)	*Aspergillus fumigatus*	2.123	*Aspergillus fumigatus*	2.134
CFA004	(+++) (A)	*Aspergillus fumigatus*	2.479	*Aspergillus fumigatus*	2.396
CFA005	(+++) (A)	*Aspergillus fumigatus*	2.453	*Aspergillus fumigatus*	2.449
CFA006	(+++) (A)	*Aspergillus fumigatus*	2.447	*Aspergillus fumigatus*	2.319
CFA007	(+++) (A)	*Aspergillus fumigatus*	2.337	*Aspergillus fumigatus*	2.246
CFA008	(+++) (A)	*Aspergillus fumigatus*	2.405	*Aspergillus fumigatus*	2.111
CFA009	(+++) (A)	*Aspergillus fumigatus*	2.518	*Aspergillus fumigatus*	2.473
CFA010	(+++) (A)	*Aspergillus fumigatus*	2.491	*Aspergillus fumigatus*	2.318
Inv1	(+++) (A)	*Aspergillus fumigatus*	2.298	*Aspergillus fumigatus*	2.136
Inv2	(+++) (A)	*Aspergillus fumigatus*	2.461	*Aspergillus fumigatus*	2.322
NEG	(-) (C)	No peaks found	< 0	No peaks found	< 0

First and second-best organism matches were referenced by the MBT Filamentous Fungi Library 2.0 (Bruker, Bremen, Germany). Each isolate was assessed in duplicate, with the first replicate reported here. Results were interpreted using a traffic light phenomenon - log scores between 2.00 - 3.00 reflect an 'A' grade (+++) and high probability of species' identification, and scores < 1.70 (-) are denoted by a 'C' grade depicting no similarity to any organism in the database. ATCC: Aspergillus fumigatus QC reference strain; NEG = uninoculated spot with Formic acid and matrix added.

Table S2: ITS gene sequencing results for isolate identification.

<table>
<tr><th>Cluster</th><th>Fungal identification</th><th>Sequences producing the most significant alignment</th><th>Blast score (bits)</th><th>Sequence identifier on GenBank (GI)</th><th>Percentage (%) identity</th><th>Query sequences</th></tr>
<tr><td>A</td><td>Aspergillus fumigatus S10</td><td rowspan="3">Small ribosomal RNA gene partial sequence; internal transcribed spacer 1, 5.8S ribosomal RNA gene, and internal transcribed spacer 2, complete sequence; and large subunit ribosomal RNA gene, partial sequence.</td><td>1081</td><td>MK267448.1</td><td>100</td><td>ATCC, CFA001, 002, 004, 005, 006, 010, Inv1, Inv2</td></tr>
<tr><td>B</td><td>Aspergillus spp. isolate CK392</td><td>1103</td><td>MK439477.1</td><td>100</td><td>CFA007, 008, 009</td></tr>
<tr><td>C</td><td>Aspergillus fumigatus strain I</td><td>1098</td><td>MK791667.1</td><td>100</td><td>CFA003</td></tr>
</table>

Sequences were analysed on the National Centre for Biotechnology Information (NCBI) Library using a standard nucleotide ‘BLAST’ algorithm, where a ‘MegaBLAST’ was performed optimising highly similar sequences with reference to the GenBank database (NCBI, USA). The top result exhibiting the highest percentage identity was exported onto MEGA X Software (MEGA: Molecular Evolutionary Genetics Analysis, University Park, PA, US) [1].

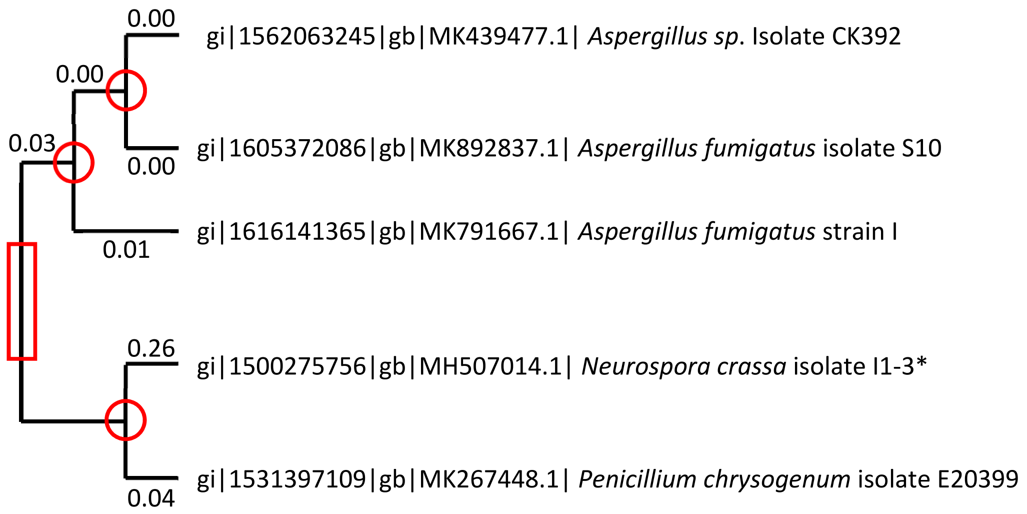

Figure S1: Neighbour-joining phylogenetic tree based on fungal species identified in the GenBank reference database. The sequencing alignments from the species identifications produced by the GenBank database were used to construct a neighbour-joining phylogenetic tree where the evolutionary distances were calculated using the Maximum Composite Likelihood method and bootstrapping with 500 replicates. * Neurospora crassa – ITS gene sequence used as control for reference of relatedness between the strains of interest. Ancestral nodes (circled, red); phylogenetic root (squared, red); gi = sequence identifier; gb = GenBank.

Reference

1. Kumar, S.; Stecher, G.; Li, M.; Knyaz, C.; Tamura, K. MEGA X: Molecular Evolutionary Genetics Analysis across Computing Platforms. *Mol. Biol. Evol.* **2018**, *35*, 1547–1549, doi:10.1093/molbev/msy096.
